# Therapeutic Dosing of Acenocoumarol: Proposal of a Population Specific Pharmacogenetic Dosing Algorithm and Its Validation in North Indians

**DOI:** 10.1371/journal.pone.0037844

**Published:** 2012-05-22

**Authors:** Saurabh Singh Rathore, Surendra Kumar Agarwal, Shantanu Pande, Sushil Kumar Singh, Tulika Mittal, Balraj Mittal

**Affiliations:** 1 Department of Genetics, Sanjay Gandhi Post Graduate Institute of Medical Sciences, Lucknow, India; 2 Cardio-Vascular and Thoracic Surgery, Sanjay Gandhi Post Graduate Institute of Medical Sciences, Lucknow, India; 3 Thoracic and Cardio-Vascular Surgery, Chhatrapati Shahuji Maharaj Medical University, Lucknow, India; Tor Vergata University of Rome, Italy

## Abstract

**Objectives:**

To develop a population specific pharmacogenetic acenocoumarol dosing algorithm for north Indian patients and show its efficiency in dosage prediction.

**Methods:**

Multiple and linear stepwise regression analyses were used to include age, sex, height, weight, body surface area, smoking status, *VKORC1* -1639 G>A, *CYP4F2* 1347 G>A, CYP2C9*2,*3 and *GGCX* 12970 C>G polymorphisms as variables to generate dosing algorithms. The new dosing models were compared with already reported algorithms and also with the clinical data for various performance measures. Odds ratios for association of genotypes with drug sensitive and resistant groups were calculated.

**Results:**

The pharmacogenetic dosing algorithm generated by multiple regression analysis explains 41.4% (p-value <0.001) of dosage variation. Validation of the new algorithm showed its predictive ability to be better than the already established algorithms based on similar variables. Its validity in our population is reflected by increased sensitivity, specificity, accuracy and decreased rates of over- and under- estimation in comparison to clinical data. The *VKORC1*-1639 G>A polymorphism was found to be strongly associated with acenocoumarol sensitivity according to recessive model.

**Conclusions:**

We have proposed an efficient north India specific pharmacogenetic acenocoumarol dosing algorithm which might become a baseline for personalised medicine approach for treatment of patients in future.

## Introduction

The human genome project completion has opened a new era of pharmacogenetics. Personalized medicine is emerging as a new therapeutic approach in clinical practice in recent times [1], [2]. Coumarinic oral-anticoagulants (COAs) like warfarin, acenocoumarol and phenprocoumon are the most frequently prescribed drugs for managing problems associated with blood coagulation in patients with atrial fibrillation (AF), heart valve replacement (HVR), deep vein thrombosis (DVT), pulmonary embolism and with patients who had undergone orthopaedic surgery [3]–[6]. More than 2 million patients are given warfarin in USA alone for preventing thromboembolism [7]. In north India, acenocoumarol/acitrom is widely used in place of warfarin. COA therapy is generally given lifelong and its dosing management is very difficult as it has a narrow therapeutic range and there are significant inter-individual as well as interethnic differences in stabilising dosages. Lower doses cause decreased efficacy in anticoagulation and higher doses increase the risk of bleeding events. Therefore, COA usage requires serial monitoring of blood coagulation by prothrombin time and international normalized ratio (INR) measurements. More importantly, the initial phase of COA therapy is very prone for clinical complications associated with over or under dosing. Deranged INR values are often observed in the first weeks of therapy and there is much higher risk of bleeding. To avoid this risk it is advised to predict the initial loading and stabilising doses of COAs [8]–[11]. At present only clinical parameters are used to predict the drug dose for anticoagulant therapy. For oral anticoagulants, the dose requirement and inter-patient variability are well known to be influenced by age, body weight, dietary vitamin K intake, concomitant disease and interacting medications [4], [12]–[14]. Genetics is a major role player in variability of drug dosage requirement to achieve therapeutic INR range [15]–[18]. More than 30 genes were found to be involved in the activity and metabolism of COAs, of which *CYP2C9* (gene coding for cytochrome P450 drug metabolizing enzyme) and *VKORC1* (gene coding for drug target enzyme) are the most important [19]. Previous studies have shown that ∼30% of the dose variance is explained by single nucleotide polymorphisms (SNPs) in *VKORC1* and another ∼12% by two non-synonymous SNPs (*2, *3) in the *CYP2C9* region [20], [21]. A genome wide association study has also revealed that *VKORC1*, *CYP2C9* and *CYP4F2* are the principal genetic factors responsible for variations in COA dose in white patients and the p values are in concordance with the fact that SNPs in *VKORC1* and *CYP2C9* regions are most significant [22]. In terms of dose variance, *VKORC1* is found to be more influencing than *CYP2C9* in some recent studies [23], [24]. However, the therapeutic drug dosage in an individual is determined by complex sets of genetic and environmental factors. Therefore, many attempts have been made in recent years to develop pharmacogenetics guided dosing algorithms based on genetic as well as clinical factors [7], [12], [17], [25]. The algorithm based dose prediction shows the importance of pharmacogenetic testing in patients who are likely to undergo COA therapy. Majority of these studies are based on warfarin dosing and not much has been explored about the pharmacogenetics of acenocoumarol and phenprocoumon. Perez-Andreu et al. have shown the importance of pre-genotyping of VKORC1, CYP4F2, CYP2C9*2 and *3 polymorphisms in patients requiring extreme doses of acenocoumarol [26]. Recently van Schie et al. have reported drug specific algorithms for acenocoumarol and phenprocoumon which are significantly different from warfarin dosing algorithms [27]. In Indian context, there is no information available in terms of genetics responsible for differences in acenocoumarol dosage requirements. Therefore, we have tried to look up in this direction and attempted to develop a pharmacogenetic algorithm to predict the stabilizing drug dosage for a better treatment of Indian patients.

## Results

### Patient characteristics

Patient characteristics are listed and compared in Table 1 between the derivation and validation cohorts. The minor allele frequencies of *VKORC1*-1639 G>A, CYP2C9*2,*3, *CYP4F2* 1347 G>A, *GGCX* 12970 C>G polymorphisms in derivation and validation cohorts were comparable. Table 1 also shows the distribution of genotypes and corresponding mean acenocoumarol doses. The *VKORC1* -1639 GG, GA and AA genotypes show daily drug dose requirements of 3.47±1.21 mg/kg,2.60±0.92 and 1.25±0.64 respectively in the derivation cohort. The respective values for the validation cohort were 3.39±1.20 mg/kg, 2.49±0.88 mg/kg and 1.47±0.22 mg/kg. There was only a single patient present each in derivation and validation cohorts with CYP2C9*2*3 genotype and they showed daily maintenance dosage requirements of 1.28 mg/kg and 2.71 mg/kg respectively. Comparatively higher drug dose requirements were observed for CYP2C9*1*1, *1*2 or *1*3 genotypes in both the derivation and validation cohorts. No significant difference was observed between patients with different allelic combinations of *CYP4F2* 1347 G>A and *GGCX* 12970 C>G polymorphisms. The trend of dosage requirement was observed for both the cohorts. All other patient characteristic were found to be comparable in both the cohorts.

**Table 1:** Patient characteristics in derivation and validation cohorts.

Variable	Derivation cohort (n = 125)	Validation cohort (n = 100)	P value
Heart valve replacement surgery (AVR/MVR/DVR)	26/82/17	18/64/18	0.63
Gender (male/female)	88/37	63/37	0.26
Age (mean ± SD)	37.45±12.27	38.05±12.85	0.72
Body weight (kg) (mean ± SD)	56.08±12.16	55.59±10.49	0.75
Height (cm)	162.59±9.67	162.19±8.98	0.75
*VKORC1* −1639 (GG/GA/AA)	85/34/6	65/32/3	0.62
*CYP4F2* 1347 (GG/GA/AA)	42/59/24	71/112/42	0.67
*CYP2C9* (*1*1/*1*2/*1*3/*2*3)	102/11/11/1	62/10/27/1	0.00
*GGCX* 12970 (CC/CG/GG)	115/8/2	97/3/0	0.22
INR (mean ± SD)	2.81±0.42	2.89±0.44	0.16
Acenocoumarol dose (mg/d) (mean ± SD)			
Overall	3.13±1.25	3.04±1.19	0.61
*VKORC1* -1639 GG	3.47±1.21	3.39±1.20	0.69
*VKORC1* -1639 GA	2.60±0.92	2.49±0.88	0.61
*VKORC1* -1639 AA	1.25±0.64	1.47±0.22	0.58
*CYP4F2* 1347 GG	2.82±1.07	2.81±0.79	0.96
*CYP4F2* 1347 GA	3.36±1.29	3.01±1.33	0.16
*CYP4F2* 1347 AA	3.08±1.36	3.51±1.23	0.30
CYP2C9*1*1	3.16±1.24	3.28±1.20	0.53
CYP2C9*1*2	3.04±1.39	2.94±1.12	0.84
CYP2C9*1*3	3.08±1.14	2.51±1.09	0.20
CYP2C9*2*3	1.28±0.00	2.71±0.00	0.38
*GGCX* 12970 CC	3.10±1.27	3.05±1.21	0.76
*GGCX* 12970 CG	3.25±0.90	2.90±0.70	0.57
*GGCX* 12970 GG	4.14±1.22	-	-

AVR/MVR/DVR: Aortic/Mitral/Double Valve Replacement.

### Pharmacogenetic dosing algorithm by multiple regression

Multiple regression analysis resulted in the following dosing algorithm: dose (mg/day)  = 3.082–0.013(smoking status, 1 for smoker and 0 for non-smoker) –0.433 (gender, 1 for male and 0 for female) –0.004(age in years) + indication(0.327 for DVR and −0.092 for AVR) +0.026(height in centimetres) +0.151(weight in kilograms) –7.660(body surface area in cm^2^) –0.862(VKORC1 GA) –2.257(VKORC1 AA) –0.049(CYP2C9*2 CT) –0.456(CYP2C9*3 AC) +0.449(CYP4F2 GA) +0.230(CYP4F2 AA) +0.245(GGCX CG) +1.055(GGCX GG) (Table 2). The coefficient of determination (R^2^) value for this equation is 41.4% (p-value <0.001). It means that 41.4% of variation in acenocoumarol dose is explained by this pharmacogenetic model.

### Pharmacogenetic dosing algorithm by linear stepwise regression

Linear stepwise regression produced a more simple equation (including the clinical and genetic factors (Table 2). The R^2^ value of this algorithm is 37% (p-value <0.001), which is the highest value produced in the stepwise regression modelling for the final step. This second dosing algorithm was found to have similar predictive trend as shown by the multiple regression equation. However, in terms of mean weekly dose and mean absolute error, the multiple regression equation was more accurate. The values of mean weekly dose and mean absolute error by stepwise regression equation were 22.02±4.72 (95% CI 21.08–22.96) and 0.71±7.86 (95% CI −0.76–2.19).

### Performance of new dosing algorithm

When compared with clinical data, the new multiple regression algorithm showed improvement in various performance measures like sensitivity (76% vs 51%), specificity (64% vs 49%), rate of overestimation (22% vs 27%), rate of underestimation (15% vs 23%), overall accuracy (63% vs 50%), accuracy in dug sensitive cases (60% vs 51%) and accuracy in drug resistant cases (72% vs 49%). The Cronbach's Alpha constant depicts the relatedness of dosage data (as a group) predicted by the new algorithm or the clinical algorithm with the actual therapeutic dosage data. This value was higher in case of new algorithm in comparison with the clinical data (0.56 vs 0.11) (Table 3).

**Table 2:** Algorithm development by multiple and linear stepwise regression analyses.

Method	Model, x variables	Regression equation	P value	R^2^ for model, %
Multiple regression	*VKORC1*, *CYP4F2*, CYP2C9*2, CYP2C9*3, *GGCX* Genotypes, weight, height, sex, age, body surface area, smoking status and indication for surgery	dose (mg/day) = 3.082–0.013 (smoking status, 1 for smoker and 0 for non-smoker) –0.433 (sex, 1 for male and 0 for female) –0.004(age) + indication(0.327 for DVR and –0.092 for AVR) +0.026(height) +0.151(weight) –7.660(body surface area) –0.862(VKORC1 GA) –2.257(VKORC1 AA) –0.049(CYP2C9*2 CT) –0.456(CYP2C9*3 AC) +0.449(CYP4F2 GA) +0.230 (CYP4F2 AA) +0.245(GGCX CG) +1.055(GGCX GG)	<0.001	41.4
Linear stepwise regression	Weight, Sex	adose (mg/day) = 1.418+0.038(weight)-0.564 (1 for male, 0 for female)	<0.001	12.5
Linear stepwise regression	VKORC1 Genotype, *weight*	dose (mg/day) = 0.755+0.896(VKORC1 GG)-1.396(VKORC1 AA)+0.033(weight)	<0.001	31.0
Linear stepwise regression	*VKORC1 Genotype*, weight, sex	dose (mg/day) = 0.192+0.879(VKORC1 GG)-1.443(VKORC1 AA)- +0.04(weight)+0.569(1 for male, 0 for female)	<0.001	34.9
Linear stepwise regression	*VKORC1*, CYP4F2 Genotypes weight, sex	bdose (mg/day) = 2.329(VKORC1 GG) +1.45(VKORC1 GA) +0.362 (CYP4F2 GA) +0.038(weight) –0.535(1 for male, 0 for female) –0.799	<0.001	37.0

a Algorithm based only on clinical variables.

b Algorithm for best fit model generated by linear stepwise regression using both clinical and genetic variables.

### Comparision of algorithms

The new multiple regression algorithm predicted better in comparison to the therapeutic dose in the aspect of standard deviation from mean weekly dose (Table 4). The mean weekly dose calculated by this algorithm was 21.26±4.82 mg/week (95% CI 20.30–22.21). This value is closest to that obtained by van Schie et al. acenocoumarol dosing algorithm, 23.56±4.67 mg/week (95% CI 22.63–24.49) [27]. The values for the algorithms by Oner Ozgon et al. [28] and Wen et al. [29] were 27.16±1.19 mg/week (95% CI 26.92–27.40) and 27.87±4.94 mg/week (95% CI 26.89–28.85). The mean value for therapeutic data of dose was found to be 21.31±8.35 mg/week (95% CI 19.65–22.97) (Table 4). The mean absolute error (MAE) for the new algorithm was 0.06±7.62 mg/week (95% CI −1.57–1.45). The MAE values were 2.25±7.86 (95% CI 0.69–3.81), 5.84±7.96 (95% CI 4.26–7.42) and 6.56±7.23 (95% CI 5.13–7.99) for van Schie et al. (acenocoumarol algorithm) [27], Oner Ozgon et al. [28] and Wen et al. [29] algorithms (Table 4).

**Table 3:** Comparision of performance of new algorithms with clinical data.

Performance measures	Multiple regression algorithm	Stepwise regression algorithm	Clinical data
Sensitivity	76%	71%	51%
Specificity	64%	58%	49%
Rate of overestimation	22%	23%	27%
Rate of underestimation	15%	13%	23%
Accuracy in all cases	63%	64%	50%
Accuracy in drug sensitive cases	60%	59%	51%
Accuracy in drug resistant cases	72%	71%	49%
Cronbach's Alpha	0.56	0.49	0.11

### Association with acenocoumarol dose

In the study subjects, *VKORC1* -1639 G>A polymorphism has the strongest association with acenocoumarol sensitivity according to recessive model (OR 4.42, 95% CI 2.44–7.99, p value<0.05). No other polymorphism was found to be significantly associated with acenocoumarol sensitive, resistant and intermediate dosing groups (Table 5).

**Table 4:** Mean weekly doses and mean absolute errors according to different algorithms.

Algorithm	Mean Weekly Dose (Standard Deviation, 95% CIConfidence Interval)	Mean Absolute Error (Standard Deviation, 95% CI Confidence Interval)
New Algorithm^a^	21.26 (4.82, 20.30–22.21)	0.06 (7.62,−1.57–1.45)
Schie et al. [27] b	23.56 (4.67, 22.63–24.49)	2.25 (7.86, 0.69–3.81)
Schie et al. [27] c	17.84 (3.11, 17.22–18.45)	−3.48 (7.36,−4.94− −2.02)
Anderson et al. [7]	40.48 (9.55, 38.58–42.37)	19.16 (9.45, 17.28–21.04)
Gage et al.[35]	37.16 (6.98, 35.78–38.54)	15.84 (9.10, 14.03–17.65)
Sconce et al. [17]	37.01 (9.56, 35.11–38.90)	15.70 (9.89, 13.74–17.66)
Wadelius et al. [21]	53.70 (11.64, 51.39–56.01)	32.39 (11.37, 30.13–34.65)
Oner Ozgon et al. [28]	27.16 (1.19, 26.92–27.40)	5.84 (7.96, 4.26–7.42)
Wen et al. [29]	27.87 (4.94, 26.89–28.85)	6.56 (7.23, 5.13–7.99)
Carlquist et al. [43]	−7.40 (19.91, -11.35– −3.45)	−28.72 (21.08,−32.9– −24.54)
Zhu et al. [44]	37.56 (8.87, 35.8–39.32)	16.25 (8.63, 14.54–17.96)
IWPC^c^ [36]	37.40 (8.46, 35.7–39.08)	16.09 (8.56, 14.39–17.79)
Miao et al. [45]	37.47 (12.08, 35.07–39.87)	16.16 (11.11, 13.96–18.36)
Ohno et al. [46]	43.64 (11.43, 41.37–45.90)	22.33 (10.48, 20.25–24.41)
Therapeutic Dose^d^	21.31 (8.35, 19.65–22.97)	

a Algorithm based on multiple regression.

b Acenocoumarol dosing algorithm, ^c^Phenprocoumon dosing algorithm.

c IWPC: International Warfarin Pharmacogenetic Consortium.

d Our cohort.

**Table 5:** Association between acenocoumarol sensitive/resistant/intermediate dose groups and polymorphisms.

Polymorphism	Acenocoumarol sensitive vs other groups, Odds ratio (95% Confidence Interval)	Acenocoumarol resistant vs other groups, Odds ratio (95% Confidence Interval)	Acenocoumarol intermediate dose vs other groups, Odds ratio (95% Confidence Interval)
*VKORC1* −1639 GA+AA	4.42 (2.44–7.99#)	0.17 (0.08–0.37)	0.91 (0.50–1.66)
*CYP4F2* 1347 GA	0.87 (0.47–1.63)	1.58 (0.82–3.03)	0.74 (0.40–1.38)
*CYP4F2* 1347 AA	1.25 (0.57–2.74)	1.52 (0.67–3.46)	0.51 (0.22–1.20)
CYP2C9*1*2	1.52 (0.63–3.64)	0.68 (0.26–1.80)	0.92 (0.36–2.35)
CYP2C9*1*3	1.93 (0.97–3.87)	0.62 (0.28–1.34)	0.77 (0.36–1.65)
*GGCX* 12970 CG	0.69 (0.18–2.66)	0.75 (0.19–2.89)	1.84 (0.54–6.26)
*GGCX* 12970 GG	0.00 (0.00–0.00)	1.98 (0.12–32.21)	2.21 (0.14–35.90)

# P-value is statistically significant.

## Discussion

It is very difficult for clinicians to predict the accurate COA dose and they depend totally on the traditional physical parameters like sex, weight, height, and age to decide the dose in advancing the therapy. Many evidences of role of genetic markers, influencing warfarin dosage, were shown by different investigators in last few years [13], [14], [30]. Genotyping of patients having *VKORC1*, *CYP2C9* and *CYP4F2* variant alleles has been shown to decrease the risk of over-anticoagulation in comparison to a fixed initial dose approach [31], [32]. Distribution of *VKORC1*-1639 A, CYP2C9*2 and CYP2C9*3 allele frequencies were found to be different for Indians when compared with selected HapMap populations [33]. The ground for population specific dosing regimens, for patients on anticoagulation, is established by such interethnic differences in allele frequencies. The *VKORC1* −1639 G>A polymorphism is present in the promoter region of the coding sequence so it decreases the enzyme expression if present in homozygous recessive state. The CYP2C9*2 and *3 polymorphisms are located in exonic region and they decrease the drug clearance as evidenced by Rettie et al. [34] in 1994. We have found only two patients with CYP2C9*2*3 genotype and they required comparatively lower drug doses than those with CYP2C9*1*1, *1*2 or *1*3 genotypes. Since the CYP2C9 enzyme is involved in metabolism of COAs, decrease in their activity will result in lower maintenance drug doses to achieve therapeutic INR.

To prevent the phenotypic extremities of COA treatment, many attempts have been made to develop pharmacogenetics guided dosing regimens for warfarin [12], [17], [21], [28], [29], [35]. The Clinical Pharmacogenetics Implimentation Consortium (CPIC) has published guidelines for the use of pharmacogenomic tests in warfarin dosing (J A Johnson et al.). Many studies have proposed warfarin dosing algorithms based on both genetic and non-genetic factors [21], [35], [36]. The pharmacogenomic algorithms in these studies have different model fitness as evidenced from the R^2^ values ranging from 34 to 63. There are only a few reports about pharmacogenetic dosing algorithms of acenocoumarol and phenprocoumon. In a recent large European cohort study, acenocoumarol and phenprocoumon algorithms were found to explain 59.4% and 49.0% variations in dose requirements respectively [27]. In the present study, we have generated a new dosing algorithm which is useful in predicting acenocoumarol doses in the Indian population. We have been able to show that 41.4% of the variability in daily maintenance acenocoumarol doses was explained by our pharmacogenetic dosing model based on age, gender, height, weight, BSA, smoking status, indication for cardiac valve replacement surgery, VKORC1 -1639 G>A, CYP4F2 1347 G>A, CYP2C9*2, *3 and GGCX 12970 C>G polymorphisms.

The dosage data generated by our pharmacogenetic algorithm displayed similar skewness as by the therapeutic maintenance dosage data. van Schie et al. [27] acenocoumarol dosing algorithm was most identical to our algorithm in terms of mean weekly dose values. The mean weekly dosage and associated standard deviation for the new algorithm and the van Schie et al. [27] acenocoumarol dosing algorithm is close to that obtained in case of therapeutic data (Table 4). The new algorithm was found to be least erroneous as the MAE was minimum in its case. van Schie et al. [27] acenocoumarol dosing algorithm was very close to our algorithm in this aspect also. Out of other 13 algorithms, 12 were derived from warfarin treated patients and 1 was derived from phenprocoumon treated patients. These algorithms show moderate to higher differences in mean weekly dose and MAE values from the therapeutic dosage data. This observation suggested drug specificity of dosing algorithms and advocated our approach. A single study from south India had used genetic and clinical parameters to develop a pharmacogenetics based dosing algorithm for warfarin and it was found to be more useful in predicting the stabilising drug doses [37]. We have not used their algorithm to predict drug doses for our patient cohort as it uses complex information of vitamin K intake and additional polymorphisms in *VKORC1* gene. Our main aim was to target most relevant markers to predict drug dose and keep the algorithm simple. Due to the difference in the type of drug (acenocoumarol in place of warfarin), genetic differences [33], [38] and dietary pattern of north Indians and south Indians, there is a need to derive a dosing regimen for acenocoumarol which is widely used in India.

The new algorithm was compared with the clinical data for different performance measures. It was significantly sensitive (76% vs 51%) than clinical data. The specificity was also increased if new algorithm was used to predict the dose (64% vs 49%). There was decrease in rate of over- and underestimation when new algorithm was compared with clinical data. Moderate increase in accuracy was observed for new algorithm in acenocoumarol sensitive cases and it was significant in acenocoumarol resistant cases (72% vs 49%). The overall accuracy was also higher for new algorithm than clinical data (63% vs 50%). The higher value of the Cronbach's Alpha constant in case of our algorithm as compared with the clinical data (0.56 vs 0.11) proves again the closer proximity of our algorithm with the therapeutic dosing.

We have found a strong association of acenocoumarol sensitivity with the heterozygocity and homozygocity for the *VKORC1*-1639 G>A change. The presence of GA or AA genotype results in very high risk of overdosing. In this condition the drug target enzyme is expressed in lower amounts so lower drug doses can achieve therapeutic INR range in early stage of treatment. Pavani et al. [37] have also reported the high risk of warfarin sensitivity for *VKORC1*-1639 G>A polymorphism. We have also looked for association of other genotypes in drug sensitive, resistant and intermediate dose groups but could not get any significant one. So we can say that *VKORC*1−1639 G>A polymorphism is the key player in drug sensitivity. We have observed presence of *CYP4F2* 1347 G>A polymorphism in the linear stepwise dosing algorithm but it was not found to be associated with any of the dose groups. This may be due to the fact that the linear stepwise regression generates a best fit model for a scalar outcome. This analysis uses both categorical as well as scalar variables as inputs. In contrast, the binary logistic regression used for associating polymorphisms with drug dose groups uses only categorical variables as inputs and it calculates the risk as odds ratios. In other words, we can say that VKORC1, CYP4F2, weight and gender explain 37% of variation in acenocoumarol dosage requirements. The R^2^ values of 41.4% and 37% in respective cases of linear stepwise regression and multiple regression algorithms suggest that VKORC1 and CYP4F2 are major contributors in acenocoumarol dosage variability. The contributions of VKORC1and CYP4F2 genotypes in overall predictive power of dosing algorithm are 21% and 3.7% respectively. There is only ∼4% increase in R^2^ value by including the other genetic factors like *CYP2C9* and *GGCX* and other non-genetic factors in multiple regression modelling. Cardiac valve replacement surgery phenotypes (double/mitral/aortic valve replacement) have ∼1.5% contribution in explaining the variability of dose requirements. This is reflected by the fact that excluding these variables from multiple regression modelling and including CYP2C9*2, *3 and GGCX 12970 C>G polymorphisms gives an algorithm with R^2^ value of 40%. This also reflects that CYP2C9*2, *3 and GGCX 12970 C>G polymorphisms explain about 3% of variability in the acenocoumarol dosage requirements.

The low frequencies of CYP2C9*2, *3 and *GGCX* 12970 C>G polymorphisms in north Indian population explain the lower contribution of these markers in variability in dosage requirements. Also, the CYP2C9*2 and *3 alleles are less frequent in the derivation cohort so their contribution is small in the algorithm. This is a limitation of our study as there may be some bias in patient selection for the two cohorts. Moreover, Loebstein et al. [39] reported that an Arg36Tyr polymorphism *VKORC1* was responsible for higher dose requirement in warfarin resistant patients and was significantly associated with higher drug dose requirement. As we have not analysed this polymorphism in our patient group, it is possible that this or some other polymorphisms might also contribute to some extent for acenocoumarol resistance.

### Conclusion

The multiple regression algorithm can be used for a more accurate prediction of acenocoumarol doses. It explains 41.4% variability in acenocoumarol dosage requirements. The linear stepwise algorithm uses genotyping of only two polymorphisms and it can be used for better cost to benefit ratio as its results are comparable with the more complex multiple regression algorithms. Polymorphisms in *VKORC1* and *CYP4F2* came out to be the principal genetic determinants explaining 37% acenocoumarol dosage variability. The less frequent CYP2C9*2, *3 and *GGCX* 12970 C>G polymorphisms in Indian population do determine the dosage variability but to an extent of ∼3% only. In comparison to other dosing models, which are mostly based on warfarin, our drug specific algorithms show more accurate acenocoumarol dosage prediction.

### Future perspective

Development of new oral anticoagulants like Dabigatran (direct thrombin inhibitor) and Rivaroxaban (direct factor Xa inhibitor) have sparked a new hope of overcoming the risk factors associated with vitamin K antagonists. These new drugs may replace the traditional oral anticoagulants in future.

However, till these new drugs are widely used, there is a need to replicate this work in a larger sample size as well as in various regions and ethnic groups of India before introduction in routine clinical practice. Improved therapy outcomes based on such studies will allow successful application of individualised dosing in clinical setting.

## Methods

### Ethics Statement

The study protocol was approved by the institutional ethical committee of Sanjay Gandhi Post Graduate Institute of Medical Sciences (SGPGIMS). The authors followed the norms of World's Association Declaration of Helsinki. All the participants gave written informed consent to participate in the study.

### Patients

The present study was carried out on the DNA samples of patients who had undergone surgery for aortic/mitral/double valve replacement and were being followed-up for regular PT-INR (Prothrombin Time-International Normalized Ratio) testing. The PT-INR testing was done every 2 weeks for each patient. A total of 7586 patients on COA therapy were screened and of which 225 patients of northern Indian origin were recruited from the outpatient department of Department of Cardiovascular and Thoracic Surgery, Sanjay Gandhi Post Graduate Institute of Medical Sciences and Department of Thoracic & Cardio-Vascular Surgery, Chhatrapati Shahuji Maharaj Medical University, Lucknow, India. The patient sample collection was done during a period of about one and half year from March 2010 to August 2011. We have divided the patients into two cohorts, namely derivation cohort of 125 patients and validation cohort of 100 patients. The selection of patients for derivation and validation cohorts was done on a random basis. All these subjects were taking maintenance dosage of acenocoumarol to achieve the INR in a therapeutic range (between 2.0 to 3.5) for at least 3 consecutive months. Patients aging less than 18 years or those suffering from diabetes, liver disease, chronic diarrheal conditions or malabsorption were excluded from the study. Patients were given dietary advice and food charts were prepared in order to avoid any interference with acenocoumarol pharmacokinetics and pharmacodynamics. Patients with interfering drug administration and showing non-compliance and were excluded from the study. Clinical data like age, body weight, height, gender, smoking habits, indication for acenocoumarol therapy, average maintenance dose and ethnicity were recorded. The average maintenance dose was calculated as mean of the dose values during the 3 month period when last two consecutive stable INR values were documented. The north Indian ethnicity of patients was decided according to their place of residence in the last three generations, food habits and mother tongue (Hindi or related languages).

### Genotyping

Blood samples were collected once in vacutainer vials coated with ethylenediaminetetraacetic acid along with the samples taken up for regular PT-INR tests. Standard salting-out method was used to isolate genomic DNA from blood samples. DNA was checked both for quantity as well as quality by gel electrophoresis and spectrophotometry using the NanoDrop Analyzer (ND-1000) spectrophotometer (NanoDrop Technologies, Wilmington, DE, USA). The ratio of absorbance of DNA at 260 and 280°nm were between 1.7 and 1.9. The checked DNA was stored at −40°C. The genotyping for *VKORC1* -1639 G>A, *CYP4F2* 1347 G>A, CYP2C9*2, *3 and *GGCX* 12970 C>G polymorphisms was done by polymerase chain reaction and restriction fragment length polymorphism (PCR–RFLP) [17], [40]–[42]. Negative (genomic DNA absent) and positive (known heterozygote) controls were used in each PCR reaction. Genotyping was repeated in ∼10% samples by different lab members to check for genotyping errors and 100% concordance was obtained. For the *GGCX* 12970 C>G polymorphism, Taqman probe-based genotyping was also performed by using the ABI prism 7900 using SNP discrimination assays designed by Applied Biosystems (ABI) (Applied Biosystems, Foster City, CA, USA).

### Statistical analyses

SPSS version 17.0 software (SPSS Japan, Tokyo, Japan) was used to perform all the statistical analyses. Descriptive statistics was used to calculate the patient characteristics. The minor allele frequencies were calculated by counting the total number of alleles for a particular SNP in all the patients and then dividing the allele with low prevalence by the allele with higher prevalence and expressed as percentage. The derivation cohort was used to generate the pharmacogenetic model which was used to predict the drug dosage in the validation cohort. The parameters used in multiple regression for deriving the pharmacogenetic model were age, sex, height, weight, body surface area (BSA), smoking status, indication for cardiac valve replacement surgery, *VKORC1* -1639 G>A, *CYP4F2* 1347 G>A, CYP2C9*2, *3 and *GGCX* 12970 C>G polymorphisms. A simpler pharmacogenetic model was also developed by linear stepwise regression using the above said variables. The genotype data was entered as 0 for absence and 1 for presence. We have not used complex dietary information of vitamin K uptake because it was too difficult to obtain that in clinical setting. Further, the patients were given dietary advice in form of charts and any noncompliance in this regard was treated as exclusion criteria. Apart from these pharmacogenetic algorithms comprising of both genetic as well as clinical factors, we have also generated an equation based only on clinical factors. This was done by putting all except the genetic factors in linear stepwise regression. We have extracted eleven algorithms through literature search which use similar parameters for dosage prediction. These algorithms were used to predict the doses based on the patient profile in the validation cohort of our study. The dosage predicted by our pharmacogenetic model was compared with the therapeutic dosage and with those predicted by the twelve algorithms. Of these 12 algorithms, 11 have been derived from patient cohorts using warfarin for anticoagulation therapy. One algorithm used patients on acenocoumarol treatment for deriving pharmacogenetic dosing algorithm. For these comparisons, we have computed the mean values of the dosage along with standard deviation (SD) and 95% confidence interval (CI) values for each algorithm. Comparison of the mean absolute error (MAE) of each algorithm was used to evaluate the performance of each algorithm. The absolute error values were calculated by deducting the actual dose from the predicted dose and their means were defined as MAE. The MAE values were calculated along with the SD and 95% CI values.

We have used SPSS software to make 3 quartiles of therapeutic drug doses of all the patients. Patients on a maintenance dose of ≤17.0 and ≥24.5 mg/week were considered acenocoumarol sensitive and resistant respectively while the patients in the range of 17.0-24.5 mg/week were assumed to be in intermediate range. As a measure of association, odds ratio values for different genotypes were calculated in both drug sensitive and drug resistant groups. The performance of our pharmacogenetic algorithms was compared with that of clinical algorithm in terms of sensitivity, specificity, rate of over and underestimation, accuracy and relatedness as a group. All these parameters were obtained by calculations by Fisher's exact test.
